# TOWARD EQUITABLE AND DESTIGMATIZING DEMENTIA: ALASKA NATIVE POPULATIONS AND CULTURALLY BASED INTERVENTIONS

**DOI:** 10.1093/geroni/igac059.191

**Published:** 2022-12-20

**Authors:** Maria Crouch, Rosellen Rosich

**Affiliations:** Yale School of Medicine, New Haven, Connecticut, United States; University of Alaska Anchorage, Anchorage, Alaska, United States

## Abstract

Alaska Native (AN) people’s incidence and prevalence of Alzheimer’s Disease and Related Dementias (ADRD) are projected to disproportionately increase in contrast to the U.S. population. This is alarming given that AN peoples experience health disparities exacerbated by prejudice, stigma, and systemic and structural inequalities. Twelve semi-structured interviews with AN Elders assessed the culturally derived meanings of memory function, loss, decline, and disease. Qualitative analyses observed eight culturally grounded themes and five interrelated and nested subthemes elucidating both the resilience and the stigmas, racism, and barriers faced by AN peoples: (1) Historical Trauma; (2) Oppression; (3) Distrust of Western Medicine; (4) Social Justice; and (5) Walking in Two Worlds. En masse historical and contemporary oppression, particularly within Western medicine, both contextualizes the present and points to the ways in which the strengths, wisdoms, and balance inherent in AN culture are imperative to the holistic health and healing.

